# Editorial: Animal nutrition and immunometabolism: from basic mechanisms to sustainable production

**DOI:** 10.3389/fvets.2026.1950498

**Published:** 2026-08-25

**Authors:** Jianyun Yuan, Panpan Cao, Ping Liu, Xiaona Gao, Xiaolong Gu, Einar Vargas-Bello-Pérez, Yu Zhuang, Guoliang Hu

**Affiliations:** 1 Jiangxi Provincial Key Laboratory for Animal Health, Institute of Animal Population Health, College of Animal Science and Technology, Jiangxi Agricultural University, Nanchang, Jiangxi, China; 2 Department of Clinical Veterinary Medicine, College of Veterinary Medicine, Yunnan Agricultural University, Panlong, Kunming, China; 3 Facultad de Zootecnia y Ecología, Universidad Autónoma de Chihuahua, Chihuahua, Mexico

**Keywords:** animals, immunometabolism, nutritional metabolic diseases, physiological functions, plant extracts

As livestock production increasingly emphasizes sustainability and efficiency, high-yielding animals face increasing metabolic demands that may challenge their physiological capacity. During critical physiological transitions, such as the perinatal period or phases of rapid growth, animals often experience negative energy balance and dysregulated lipid metabolism. These metabolic disturbances can lead to systemic dysfunctions, including hepatic steatosis and subclinical ketosis (SCK) (1). Traditionally regarded primarily as energy substrates or structural components, nutrients and their metabolites are increasingly recognized as important modulators of cellular signaling and immunometabolic processes. Growing understanding of immunometabolic regulation has stimulated interest in natural plant extracts and their bioactive compounds as potential modulators of metabolic and immune pathways. These compounds may therefore provide complementary strategies for maintaining metabolic homeostasis and supporting animal health.

The present Research Topic highlights the mechanisms and potential applications of phytogenic compounds and related nutritional strategies for alleviating metabolic disorders in livestock. By linking molecular signaling pathways to physiological and production outcomes, this Research Topic offers insights into how phytogenic compounds and related nutritional interventions influence host-microbiome interactions, regulate nuclear receptor signaling, and modulate cellular energy metabolism.

At the molecular level, regulation of lipid and energy metabolism represents a central component of metabolic homeostasis. Jia et al. reported that activation of the farnesoid X receptor (FXR) reduced free fatty acid (FFA)-induced triglyceride accumulation in hepatocytes and attenuated oxidative stress, as indicated by decreased levels of reactive oxygen species (ROS) and hydrogen peroxide (H_2_O_2_). These findings are consistent with the established role of FXR in regulating bile acid and lipid metabolism (2, 3) and suggest that FXR may represent a potential target for further investigation in hepatic steatosis. Beyond receptor-mediated regulation, metabolic interventions may also contribute to the restoration of energy homeostasis. Xue et al. reported that thiamine supplementation improved energy metabolism and promoted β-hydroxybutyrate (BHBA) clearance. BHBA is a major circulating ketone body and an important biomarker of subclinical ketosis. Its reduction was accompanied by improvements in SCK-associated metabolic abnormalities. Collectively, these findings highlight the potential value of improving metabolic efficiency to restore energy homeostasis.

Beyond host metabolic regulation, the gut microbiota represents an important interface between diet and host physiology. Wen reported that gut microbiota-derived tryptophan metabolites, particularly indole derivatives, can act as microbial signaling metabolites that influence host responses. Through signaling involving the aryl hydrocarbon receptor (AhR) and pregnane X receptor (PXR), these metabolites may contribute to immunometabolic regulation, maintenance of mucosal barrier integrity, and systemic immune homeostasis (4–6). These findings also support growing interest in dietary and naturally derived strategies that regulate inflammatory responses and signaling associated with the microbiota (7–9). Expanding on this systemic perspective, Zhang et al. reported that biochar produced from apple wood was associated with systemic physiological changes. Biochar supplementation was associated with changes in circulating immune-related pathways, including IL-17 signaling and complement responses. These changes were accompanied by improved indicators of disease resistance and feed efficiency in growing pigs.

Translating phytogenic compounds and related nutritional interventions from experimental research into commercial livestock production requires careful evaluation of dose, inclusion level, efficacy, and safety. Xiang reported distinct dose-dependent responses to resveratrol in broilers. Supplementation at 250 mg/kg reduced abdominal fat deposition, whereas 1,000 mg/kg improved antioxidant capacity and growth performance. Li et al. further highlighted the importance of appropriate inclusion levels when incorporating agricultural byproducts into animal diets. While a mixed fermented silage of cotton stalks and apple pomace enriched rumen fiber-degrading microbial populations, using it as a total replacement for traditional corn silage reduced ruminal volatile fatty acid concentrations (VFAs) and deteriorated carcass characteristics. At the molecular level, Jia et al. reported that FXR suppression aggravated FFA-induced lipotoxicity, highlighting the need for precision when designing interventional strategies. Collectively, these findings emphasize that successful nutritional interventions require careful optimization of dose, inclusion level, and molecular targets.

Together, these studies suggest that phytogenic compounds and related nutritional strategies may offer complementary approaches for managing metabolic disorders associated with nutritional imbalance. By revealing how these interventions modulate host-microbiome interactions, nuclear receptor signaling, and cellular metabolism, these findings provide a mechanistic basis for more precise nutritional strategies in livestock. Further progress in precision animal nutrition will benefit from integrating systems biology with advanced multiomics approaches. Such an integrated approach may facilitate the characterization of complex metabolic networks and help define effective and safe dose ranges for plant-based nutritional interventions. These advances may ultimately support the appropriate application of such strategies in livestock production (10).
